# Different Trends in Microbial Contamination between Two Types of Microfiltered Water Dispensers: From Risk Analysis to Consumer Health Preservation

**DOI:** 10.3390/ijerph16020272

**Published:** 2019-01-18

**Authors:** Luna Girolamini, Jessica Lizzadro, Marta Mazzotta, Matteo Iervolino, Ada Dormi, Sandra Cristino

**Affiliations:** 1 Department of Biological, Geological, and Environmental Sciences, University of Bologna, via San Giacomo 12, 40126 Bologna (BO), Italy; luna.girolamini2@unibo.it (L.G.); jessica.lizzadro2@unibo.it (J.L.); marta.mazzotta2@unibo.it (M.M.); matteo.iervolino2@unibo.it (M.I.); 2 Department of Medical and Surgical Science, University of Bologna, via San Giacomo 12, 40126 Bologna (BO), Italy; ada.dormi@unibo.it

**Keywords:** microbiological contamination, microfiltered water dispensers (MWDs), sanitation measures, drinking water safety plan (DWSP)

## Abstract

The use of microfiltered water dispensers (MWDs) for treatment of municipal water is increasing rapidly, however, the water quality produced by MWDs has not been widely investigated. In this work a large-scale microbiological investigation was conducted on 46 MWDs. In accordance with Italian regulations for drinking water, we investigated the heterotrophic plate counts at 36 and 22 °C for indicator bacteria and pathogenic bacteria, such as Enterococci, *Pseudomonas aeruginosa*, *Escherichia coli* and *Staphylococcus aureus*. Two different MWDs were compared: Type A with Ag^+^ coated carbon filter and two ultraviolet (UV) lamps, and Type B with a carbon filter and one UV lamp. For each type, the contamination of the input and output points was analyzed. Our findings showed that MWDs are a source of bacteria growth, with output being more contaminated than the input point. Type B was widely contaminated for all parameters tested in both sampling points, suggesting that water treatment by Type A is more effective in controlling bacterial contamination. MWDs are critical devices for water treatment in term of technologies, intended use, and sanitization procedures. The adoption of an appropriate drinking water safety plan associated with clear maintenance procedures and periodic environmental monitoring can ensure the safe and healthy operation of these devices.

## 1. Introduction

The use of different sources to obtain water for human consumption instead of tap water or bottled water has been continuously increasing. One of these new sources is the microfiltered water dispenser (MWD). Since their introduction, MWDs have been introduced in industrial companies, university campuses, commercial buildings, etc. [1]. MWDs offer an alternative to bottled water, overcoming and even eliminating drawbacks that worsen the environmental impact of these products, such as the disposal of the container materials (e.g., plastic) [2]. MWDs are connected to the municipal water supply and can produce room temperature, chilled, or sparkling water. They are called microfiltered water dispensers because they contain filtration systems, such as activated carbon filters, sometimes associated with a membrane coated with Ag^+^ ions to produce a bacteriostatic effect [3,4]. Activated carbon filters are the most common system used to reduce the undesirable tastes and odors and remove organic and inorganic contaminants (e.g., humic acid, clays, chlorine, and residue by-products). The filtration systems are often associated with bactericidal ultraviolet (UV) lamps that act when water passes through the pipe associated with the UV lamp [5]. UV lamps are inserted inside the MWDs or at the water output points (nozzles) and are generally used for the destruction of airborne or surface microorganisms [6,7,8]. However, its germicidal effectiveness can be hindered by organic matter such as soil and biofilm [9]. The bacteria, amoebae, etc., present in the water can form an adherent biofilm inside the water conducts, which can increase the risk of water contamination [10,11].

The term “biofilm” describes a growth pattern where opportunistic pathogens, such as *Pseudomonas aeruginosa* and *Legionella*, which can negatively affect human health [12,13,14], thrive immersed in fluid and aggregate in a self-produced extracellular polymeric substance [12,15,16]. Therefore, bacteria growing within biofilms are more resistant to antimicrobial agents than planktonic cells of the same species [17,18].

To prevent contamination and biofilm formation and guarantee satisfactory water quality produced by MWDs, different sanitization procedures are applied following the standards prescribed by European and Italian regulations for drinking water [5,19,20,21,22]. However, few studies have been published on the microbiological quality of the water supplied from MWDs [23,24,25], despite the general increase in the use of these devices and the improvements in the technologies applied to water treatment. The reasons for the limited information about MWDs is probably associated with common use of the devices and a lack of a control performed by health authorities, who sometimes are not notified about their installation and use.

In Italy, it is mandatory for canteens and restaurants to notify the Public Health Authorities [26] about the use of MWDs, resulting in limited knowledge about the water quality produced by MWDs and the risk that can occurs thought water consumption by consumers.

Thus, the aim of this study was to evaluate the microbiological quality of the water supplied by two different types of MWDs located in different areas of an industrial site, called Type A and Type B based on their different water treatment technologies. Type A is characterized by a carbon filter, being Ag^+^-coated, and two UV lamps and Type B is based on a carbon filter and one UV lamp. To understand the contamination dynamics, we compared two sampling points in MWDs for each type: the input vs. the output. Then, we studied the contamination differences and focused on the technologies used for water treatment, the maintenance, and sanitization procedures performed. The microbiological quality of the water was tested through the determination of a typical indicator bacteria—heterotrophic plate counts (HPCs) at 36 °C and 22 °C—and pathogenic bacteria, such as Enterococci, *Pseudomonas aeruginosa*, *Escherichia coli*, and *Staphylococcus aureus*, according to the Italian regulations for MWDs and drinking water [5,19].

## 2. Methods

### 2.1. MWD Characteristics and Operating Measures

**Figure 1:**
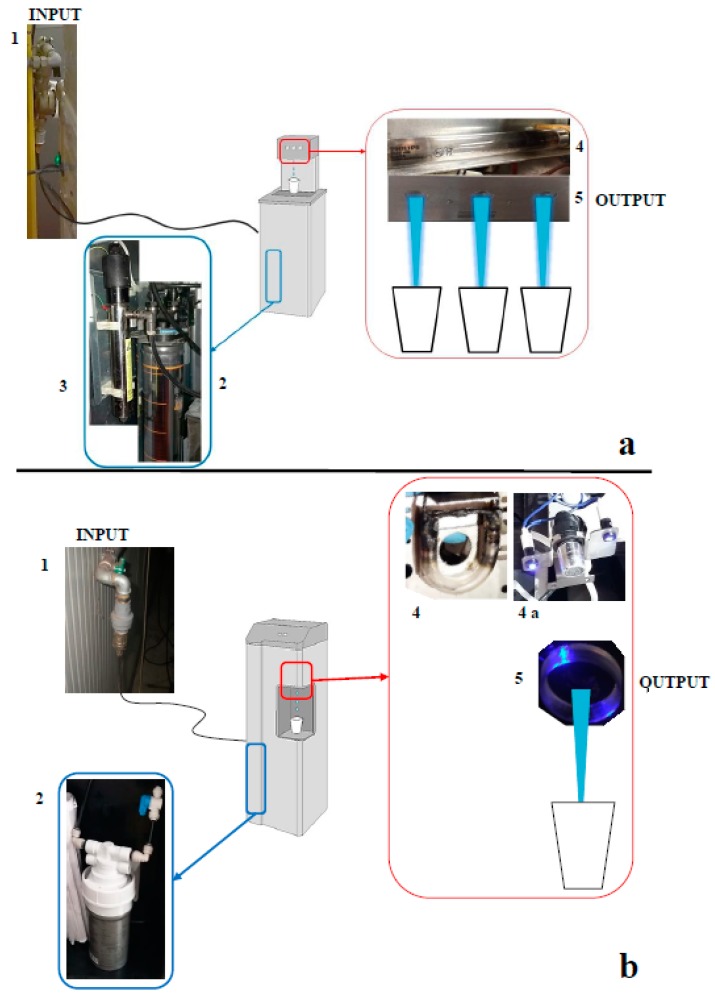
Schematic representation of MWDs (microfiltered water dispensers): (**a**) Type A and (**b**) Type B. (1: Input from municipal water, 2: carbon filter, 3: ultraviolet (UV) inner lamp, 4: UV lamp at water supply point, 4a: UV lamp, without glass and water supply point (output), and 5: water supply points (output)).

From 2015 to 2017, MWDs of two different types (called Type A and Type B for privacy reasons) were installed at a metalworking industrial site near Bologna, Italy. Type A, composed of a carbon filter coated with Ag^+^ and two UV lamp, was installed inside the canteens. The carbon filter, with a pore size of 0.5 μm, reduces tastes and odors, various organic and inorganic substances (e.g., humic acids, clays, chlorine, etc.), as well as disinfects byproducts in municipal water, and does not retain bacteria or viruses, while the Ag^+^ coating produces a bacteriostatic effect [5,25]. One low-pressure UV lamp (inner lamp) is located in a central body of the device after the carbon filter, and another low-pressure UV lamp consisting of a small quartz tube is placed above the supply point (output), with powers of 2.6 and 0.9 W, respectively. This type of MWD supplies chilled water, water at room temperature, and sparkling water as per the consumer’s request via three different pipelines feeding three supply points (Figure 1a).

Type B was installed in the common or private spaces within the industrial site (e.g., work area, coffee break area, offices, and recreational points). Type B is composed of a carbon filter with a pore size of 0.5 μm, without a Ag^+^ coating, and a single low-pressure UV lamp at the water supply point (output). The lamp consists of a small glass tube encircling the supply point with a power of 1.5 W. Type B provides water at room temperature and chilled water via one supply point (Figure 1b).

Both filters are “point of use” types used to treat water for drinking, meaning that they are connected to a main potable water network (municipal water) through a dedicated pipe containing a removable filter with pore size of 1 mm to remove particulate matter that can arrive from the water network. The capacity of MWDs are approximately 120–180 L/h for Type A and 20–28 L/h for type B.

The technical characteristics of MWDs were provided in the manufacturers’ manuals and conformed to the Italian regulations [5]. The purpose of the regulation is to guarantee that the treatments do not compromise the water quality of water already suitable under the health profile, that the processing equipment guarantees the claimed effects, and that complete information on the effects of treatments is provided to the consumer.

### 2.2. Collection of Water Samples

Following the drinking water safety plan (DWSP) for industrial site according to the World Health Organization (WHO) and Italian regulation for drinking water [19,27], the MWDs were monitored every six months to analyze water quality. From October 2015 to November 2017, a total of 46 MWDs were sampled. Out of 46 MWDs, 11 were categorized as Type A and 35 as Type B according to their technical characteristics. The sampling was repeated when the results were outside of legislation limits and disinfection procedures were undertaken for a total of 45 samples for Type A and 140 for Type B. For each MWD, 500 mL of water at room temperature were collected each from the input (a point connected to the municipal water distribution system) and output (point of water supply). The samples were collected according to EN ISO 19458 [28] via the post-flushing modality (running water for 1 min maximum) in sterile polytetrafluoroethylene (PTFE) bottles and maintained at 4 °C until the analysis, which was performed the same day.

### 2.3. Microbiological Analyses

All water samples were analyzed for indicator bacteria using heterotopic plate counts (HPCs) at 36 and 22 °C, and for the presence of pathogenic bacteria including Enterococci, *P. aeruginosa*, *E. coli*, and *S. aureus* in accordance with Italian regulations for drinking water [19]. To examine whether the samples met the criteria for drinking water for human consumption, all analysis were performed according to the reference standard methods. The HPC analysis was performed according to UNI EN ISO 6222:2001 [29], using the standard plate method on tryptic glucose yeast agar (PCA; Biolife, Milan, Italy). Analysis of Enterococci was carried out using the standard membrane filter technique according to ISO 7899-2:2000 [30] using Slanetz Bartley agar (Biolife). The analysis for *P. aeruginosa* was carried out using the standard membrane filter technique, according to UNI EN ISO 16266:2008 [31] using Pseudomonas selective agar (PSA; Biolife). The *E. coli* bacteria were analyzed using the standard membrane filter technique according to UNI EN ISO 9308-1:2017 [32] using chromogenic coliform agar (CCA; Biolife). *S. aureus* contamination was detected using the standard membrane filter technique according to ISO 16140-2:2016 [33] using Brilliance Staph 24 agar (BPA, ThermoFisher Scientific, Oxoid Ltd., Basingstoke, UK).

Suspected colonies for each species grown on the selective media were sub-cultured and identified using a Crystal Enteric/Non-Fermenter ID kit (Crystal E/NF) or BBL Crystal Gram Positive ID kit (Crystal GP), both obtained from Becton Dickinson (Cockeysville, MD, USA), according to the manufacturer’s instructions [34].

The results for HPCs at 36 °C and 22 °C are expressed as mean Log cfu/mL, and the data for Enterococci, *P. aeruginosa*, *E. coli*, and *S. aureus* are expressed as mean Log cfu/100 mL according to regulation limits [19]. The samples under regulation limits were considered negative.

### 2.4. Statistical Analysis

Bacteriological data were converted into log_10_ x (Log x) to normalize the non-normal distribution of the data, which are expressed as mean concentration ± standard deviation (SD) of positive samples. The results were then analyzed using Student’s t-test (Stata 10 Data Analysis and Statistical Software; StataCorp LP, College Station, TX, USA) and Fisher’s exact test (SPSS 23 Data Analysis and Statistical Software; version for Windows; IBM, Chicago, IL, USA). *p*-values (*p*) < 0.05 were considered statistically significant.

## 3. Results

From 46 MWDs, we collected and analyzed a total of 185 samples. From this, 93 samples had a positive value for one or more parameters (over the regulation limits) in one sampling point (input or output) or in both sampling points (39 samples of Type A and 54 samples of Type B). As mentioned above, the total contamination for a single MWD was calculated by averaging the input and output value, when one or both points exceeded the regulation reference value [19].

The data analysis about total MWDs samples revealed that 93/185 (50.3%) were contaminated for one or more microbiological parameters over the regulation limits [19].

**Figure 2:**
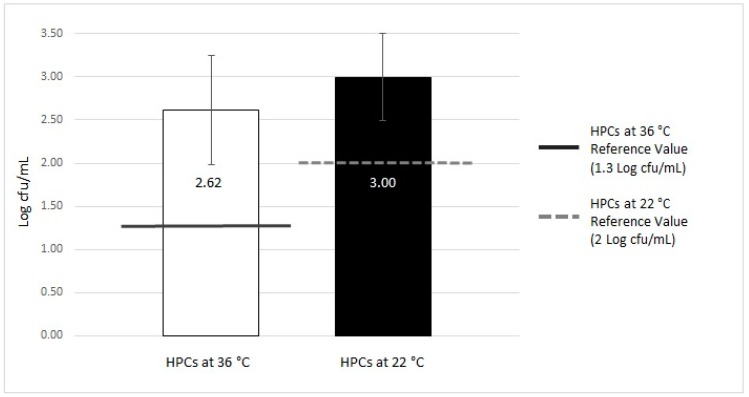
Microbiological contamination of total of samples of MWDs (microfiltered water dispensers): analysis of HPCs (heterotrophic plate counts) at 36 °C and 22 °C analysis.

**Figure 3:**
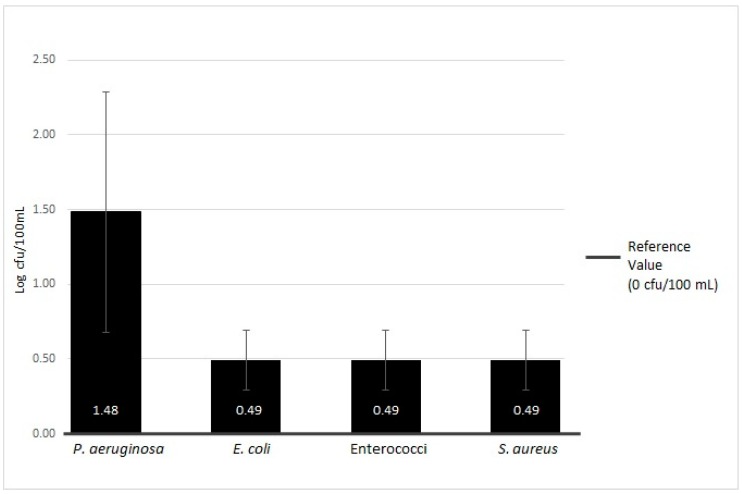
Microbiological contamination of total of samples of MWDs: Enterococci, *Pseudomonas aeruginosa*, *Escherichia coli*, and *Staphylococcus aureus.*

The details of the microbiological contamination level are shown in Table 1. The trends in contamination with respect to reference regulation values are presented in Figure 2 and Figure 3.

The data analysis of the sampling points (input and output water) revealed all positive samples were contaminated at both the sampling points for one or more microbiological parameters. In particular, we found a higher contamination level over the regulation limits [19], at the output for HPCs at 36 °C and 22 °C, *P. aeruginosa* and other microorganisms. The contamination level details are shown in Table 1.

For HPCs at 36 °C, 44.09% (41/93) of samples were positive with a mean value of 2.13 ± 0.50 Log cfu/mL, and 60.22% (56/93) with a mean of 2.90 ± 0.65 Log cfu/mL for input and output water, respectively. This difference was statistically significant (*p* = 0.003). Regarding HPCs at 22 °C, 6.45% (6/93) of samples were positive for input water (2.66 ± 0.56 Log cfu/mL), whereas 35.48% (33/93) were positive for output water, (3.06 ± 0.60 Log cfu/mL). No significant difference was observed between the two groups (*p* = 0.45).

In relation to the analysis of *P. aeruginosa*, a greater percentage (21.51%, 20/93) of samples were positive, with a mean value of 1.48 ± 0.80 Log cfu/100 mL for output water. There was a statistically significant difference in the output water samples (*p* < 0.001), as the input water did not show the presence of microorganisms. The results concerning the contamination by other pathogenic microorganisms (Enterococci, *E. coli*, and *S. aureus*) showed that 3.23% (3/93) of only the output water samples were positive (0.49 ± 0.20 Log cfu/100 mL), with a statistically significant difference (*p* < 0.001).

**Figure 4:**
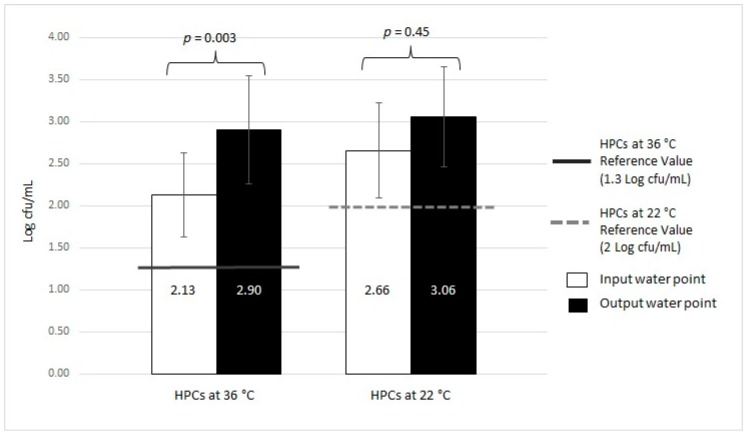
Microbiological contamination of MWDs (input vs. output) based on HPCs analysis at 36 °C and 22 °C.

**Figure 5:**
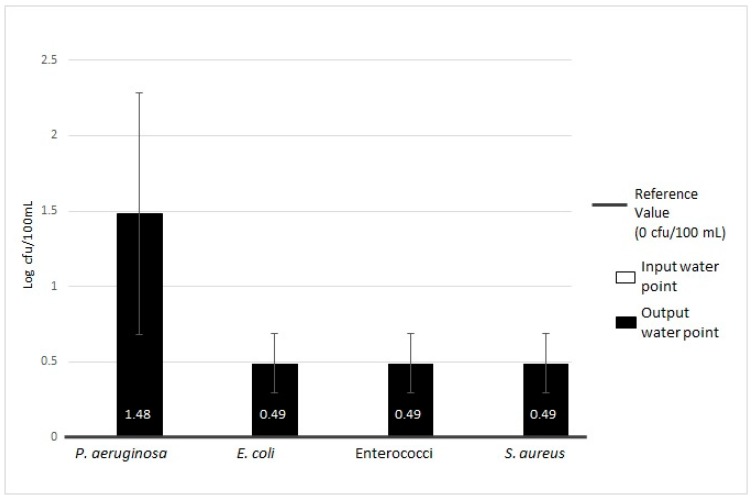
Microbiological contamination of MWDs (input vs. output)—Enterococci, *Pseudomonas aeruginosa*, *Escherichia coli*, and *Staphylococcus aureus*.

The trends in contamination with respect to the reference Italian regulation value for drinking water [19] are represented in Figure 4 and Figure 5.

We also analyzed the microbial contamination in relation to different water treatment technologies, i.e., Type A (double UV lamp) and Type B (one UV lamp) (Table 2). The results were obtained by analyzing 11 Type A and 35 Type B MWDs. As mentioned above, the total contamination for a single MWD was calculated by averaging the input and output value, which is expressed as mean ± SD.

For Type A devices, as shown in Table 2, we found that 51.28% of the MWDs (20/39) were contaminated with HPCs at 36 °C (2.33 ± 0.56 Log cfu/mL), above the limit allowed by the Italian regulation for drinking water [19]. The same trend was found for the levels of HPCs at 22 °C, where the percentage of contaminated devices was 15.38% (6/39) with a mean value of 2.51 ± 0.44 Log cfu/mL. We found *P. aeruginosa* in 2.56% (1/39) of samples (0.90 Log cfu/100 mL), whereas the samples from Type A devices were not positive for other pathogenic microorganisms, including Enterococci, *E. coli*, and *S. aureus*.

With respect to the Type B MWDs, 98.15% (53/54) and 51.85% (28/54) of samples displayed levels of HPCs at 36 °C and HPCs at 22 °C (2.72 ± 0.68 Log cfu/mL and 3.10 ± 0.49 Log cfu/mL, respectively) above the Italian regulation limit for drinking water: Legislative Decree n. 31 (02.02.2001) [19]. Of the Type B samples, 35.19% (19/54) were positive for *P. aeruginosa* (1.51 ± 0.81 Log cfu/100 mL) and 5.56% (3/54) of the samples were positive for other pathogenic microorganisms (0.49 ± 0.20 Log cfu/100 mL). The differences found between Type A and B were statistically significant for HPCs at 36 °C and HPCs at 22 °C (*p* = 0.0175 and *p* = 0.0088, respectively), other than for *P. aeruginosa* (*p* < 0.001) and for other pathogenic bacteria (Enterococci, *E. coli* and *S. aureus*) (*p* < 0.001).

**Figure 6:**
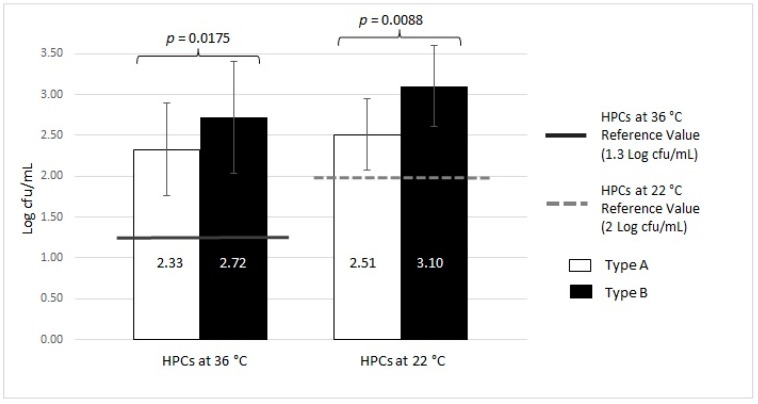
Microbiological contamination of total Type A and total Type B samples (MWDs): analysis of HPCs at 36 °C and 22°C.

**Figure 7:**
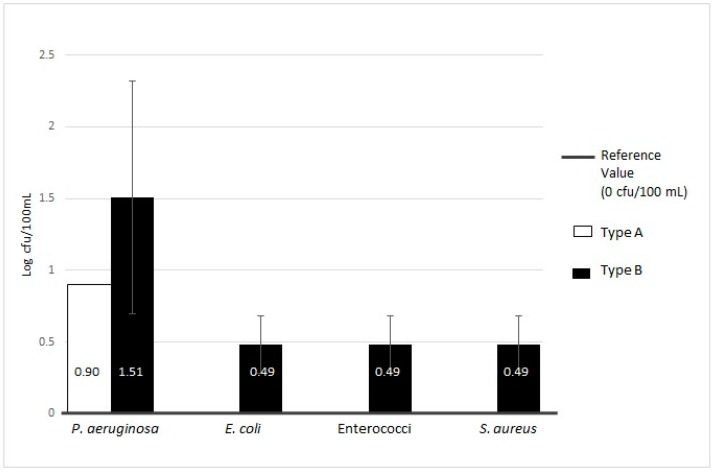
Microbiological contamination of total samples of Type A vs. Type B (MWDs) for Enterococci, *Pseudomonas aeruginosa*, *Escherichia coli*, and *Staphylococcus aureus*.

The trends in contamination with respect to the reference regulation value are presented in Figure 6 and Figure 7.

Finally, we analyzed the contamination found in both MWD types with respect to the two different water sampling points: input and output (Table 2). In Type A, as shown in Table 2, 43.59% (17/39) and 25.64% (10/39) were positive samples, with mean values for HPCs at 36 °C of 2.16 ± 0.47 Log cfu/mL and 2.67 ± 0.38 Log cfu/mL in the input and output water, respectively. Statistical analysis showed a significant difference in HPCs at 36 °C between input and output samples in Type A devices (*p* < 0.001). A greater percentage of samples (12.82%, 5/39) were positive with respect to HPCs at 22 °C in the output water compared to the input water samples (5.13%, 2/39). The mean values were 2.86 ± 0.28 Log cfu/mL and 2.58 ± 0.62 Log cfu/mL for input and output, respectively. The statistical analysis showed a significant difference in HPCs at 22 °C between input and output samples in Type A devices (*p* = 0.013).

The output water of the Type A MWDs displayed the presence of *P. aeruginosa* in 2.56% of samples (1/39), with a mean value of 0.90 Log cfu/100 mL. In this case, there is no standard deviation, as we only recorded one value. The statistical analysis showed a significant difference in the *P. aeruginosa* content in the output samples from Type A devices (*p* < 0.001). The data on the total contamination in the Type A devices with respect to other pathogenic microorganisms, including Enterococci, *E. coli*, and *S. aureus*, revealed that both input and output water were negative.

In the case of HPCs at 36 °C in Type B MWDs, the percentage of positive samples was greater in the output water (85.19%, 46/54) than in the input water samples (44.44%, 24/54). The output samples had a mean HPC at 36 °C of 2.97 ± 0.67 Log cfu/mL, whereas the input samples displayed a mean of 2.11 ± 0.53 Log cfu/mL. Statistical analysis showed a significant difference between input and output samples with respect to HPCs at 36 °C in Type B devices (*p* = 0.006). We found that 7.41% (4/54) of samples in the input water in Type B devices were positive for HPC at 22 °C with a mean value of 2.56 ± 0.68 Log cfu/mL, whereas 51.85% (28/54) of output water samples were positive, with a mean value of 3.14 ± 0.56 Log cfu/mL. Statistical analysis did not show a significant difference between HPCs at 22 °C in the input and output samples of Type B devices (*p* = 0.065).

For *P. aeruginosa* contamination, 35.19% (19/54) of samples were positive only in the output water (1.51 ± 0.81 Log cfu/mL), but there were no positive samples in the input water. Statistical analysis showed a significant difference between input and output samples concerning *P. aeruginosa* in Type B devices (*p* = 0.029). Furthermore, 5.56% (3/54) of the samples were positive for other pathogenic microorganisms, including Enterococci, *E. coli*, and *S. aureus*, with a mean value of 0.49 ± 0.20 Log cfu/100 mL. These results were obtained only in the output water and the difference in the other microorganisms in Type B devices was significant (*p* < 0.001).

There were no statistically significant differences in HPCs at 36 °C and HPCs at 22 °C of the input water samples between Type A and Type B (*p* = 0.6033 and *p* = 0.7055, respectively). However, there were statistically significant differences in the HPCs at 36 °C and HPCs at 22 °C in the output water samples between Type A and Type B (*p* = 0.0422 and *p* = 0.0261, respectively).

**Figure 8:**
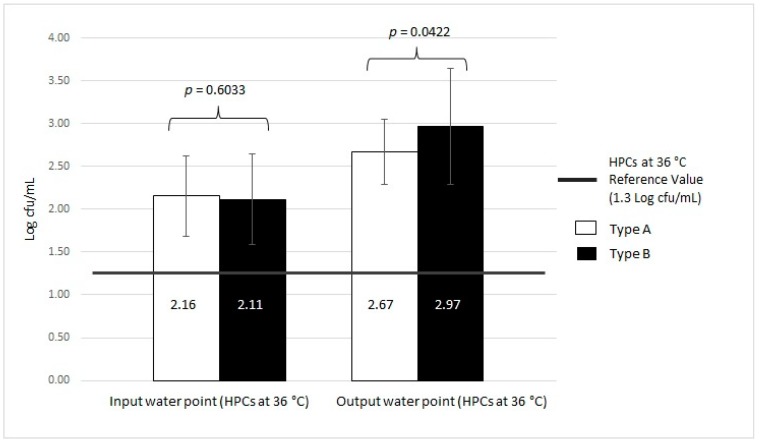
Microbiological contamination in Type A and Type B input vs. output samples using HPC analysis at 36 °C.

**Figure 9:**
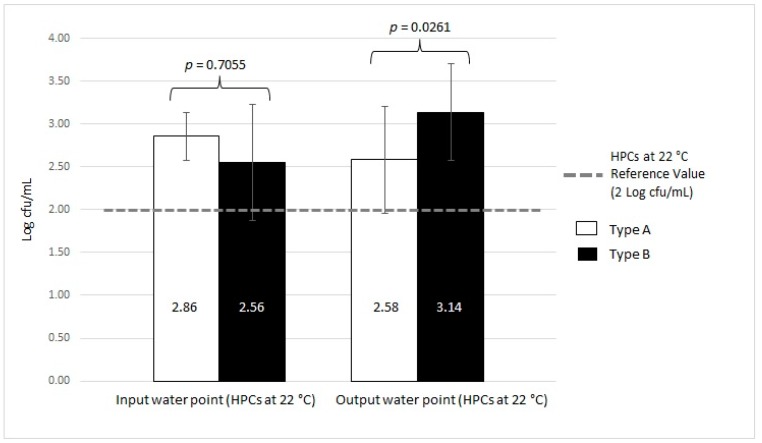
Microbiological contamination in Type A and Type B input vs. output samples using HPC analysis at 22 °C.

The trends in contamination with respect to the reference regulation value obtained from the Italian regulation for drinking water [19] are shown in Figure 8 and Figure 9.

Given these results, the disinfection and maintenance procedures applied by industrial stakeholders were changed and/or implemented as summarized in Table 3.

## 4. Discussion

MWDs are increasingly being used both within private homes and in places of aggregation, such as offices, canteens, and university campuses. MWDs satisfy the needs of the consumers for good quality water without taste, odor, or microbiological contaminants. To satisfy these demands, appropriate cleaning and sanitation measures must be implemented to ensure safe water is produced by the use MWDs, following an appropriate drinking water safety plan (DWSP), as suggested by the Italian regulation for drinking water and its new revision [35], transposed from the Commission Directive (EU) 2015/1787 [36].

This study aimed to determine the quality of drinking water produced by MWDs located in different areas of an industrial site according to the DWSP and the worker safety directive [37]. To the best of our knowledge, this study is the first long-term (two years) and large-scale monitoring of drinking water quality in an industrial site with a large number of MWDs (*n* = 46). The contamination was studied for each MWD at two points, input and output, to assess the pathway of microbial colonization in these devises. The results obtained were correlated with the technologies used by devices: a carbon filter with Ag^+^ coating and double UV lamp (Type A), and a single carbon filter without Ag^+^ coating, plus UV lamp at the output point (Type B); implemented following the manufacturers procedures and ordinary use by the consumers.

From a careful analysis of the results obtained during the monitoring of MWDs, we confirmed a high contamination level of HPCs at 36 °C, HPCs at 22 °C, and some pathogenic bacteria, as already verified by previous studies [1,2,21,38]. The analysis of results obtained comparing the two sampling points showed that the output points of MWDs are more frequently contaminated by all microbiological parameters respect to the input points.

In comparing the type of technologies used in MWDs to produce water, Type A vs. Type B, we found a significant difference in HPCs at 36 °C and 22 °C between the devices as well as in the pathogenic bacteria, with Type B devices being always more contaminated than Type A. The data acquired from both sampling points (input and output) confirmed that output samples from Type B devices were more contaminated compared to the output point of Type A, for all parameters tested. By contrast, no statistical differences were found between samples collected from input of the two types of devices, confirming that the municipal water distribution network is less affected by bacterial colonization.

We can explain the different contamination difference between the MWDs based on three aspects. The first is the type of device. Type B MWDs produce microfiltered water by carbon filtration to absorb taste and odor, and reduce the residues of chlorination without Ag^+^ coating and with one low-pressure UV lamp, situated at the supply point, replaced or cleaned by manufacturer once a year, introduced to develop the disinfection activity [39]. We observed how this lamp in the Type B device consists of a small glass tube encircling the supply point with radiation that is too far from the water flow, which is one of the requirements for disinfection [40]. During our inspection, we found the lamp broken and non-functional although the internal light was switch on, reducing the disinfection activity. The two lamps in Type A device, with an inner flow around a large quartz tube and a second irradiation due to another lamp being attached to the nozzles (output), ensure the best performance in terms of bacteria inactivation, contributing to lower bacteria colonization.

The new protocol for the replacement and cleaning of lamps was created to minimize problems due to bacterial contamination, including an increase in the amount and frequency of cleaning procedures on glass/quartz tubes, replacing UV lamps every six months by manufacturer, and monthly control by the industry maintenance stakeholders.

Another important point is the volume of water produced by dispensers. For example, Type A devices produce a larger volume of water (approximately 120–180 L/h with a water consumption of 8000–20,000 m^3^/year/MWD) and are located in canteens, where the consumers usually fill carafes with about two or more liters during lunch time. Type B devices were positioned in a large space located in the industrial site (work area, offices, and recreational points) where the water consumption is lower. The different volume of water produced (approximately 20–28 L/h with a water consumption of 500–600 m^3^/year/MWD) can increase the duration of water stagnation and increase the biofilm formation [41]. In distribution networks, uncontrolled detachment of biofilm, should be common, due to non-continuous consumption of drinking water, and therefore could lead to variable concentration of HPCs bacteria in the water. High HPCs measurements within building plumbing systems may also be caused by bacterial regrowth or by contamination events (pipeline breaks or renovation work on water plumping system) in addition to consumers’ behaviours [42]. The increase in flushing time implemented during the study (every morning, and every Monday after the weekend) increase the volume of water dispensed and minimize stagnation duration, especially when the devices are scarcely used, such as over the weekend or on holidays.

The second aspect is linked to the disinfection of devices, which was previously performed by manufacturers only during filter removal (once a year), consisting of a continuous treatment for 10 min with hydrogen peroxide solution (3%, *v/v*) that was injected in the device by a pump, and circulation in the devices, as described in Table 3. The flow of disinfectant across the device probably did not permit sufficient contact time for the activation of peroxide and achievement of the bactericidal effect [43,44]. Our study also revealed that some MWDs components are difficult to sanitize and this prevents hygienic maintenance of the machine. For example, the supply point is often located internally and is not easily assessable; when cleaning procedures are conducted, this part of device cannot be disassembled. Thus, the contact time between device and disinfectant is insufficient. Hence, it is desirable to install these devices with removable nozzles that can be disassembled and cleaned. According to the manufactures, we changed the disinfection protocol, increasing the disinfectant contact time to at least 20 min, rewashing the device before and after disinfection, the disassembling of nozzles to permit a descale, and disinfection treatment. This new protocol is introduced in DWSP procedures.

The third reason is linked to the position of these devices inside the company. The Type B MWDs were often present in aggregation areas inside productive spaces with low ventilation, low air exchange, and accumulation of pollutants from the machines. The consumers were likely to use the device with work gloves and/or without washing hands, often filling old plastic or glass bottles directly at the output nozzles or making direct contact with the nozzles with dirty hands or mouths. The presence of some pathogenic bacteria in the output samples, such as Enterococci, *P. aeruginosa*, *S. aureus*, and *E. coli*, confirm the human origin of this contamination. To avoid this problem, the industry introduced the communication of these risks to consumers.

A general reason for MWD contamination is the lack of clear and adequate maintenance procedures in the manuals provided by the manufacturers. In many cases, there are no specific indications about maintenance and sanitation procedures resulting in a series of incorrect behaviors by the operators, leading to poor water quality. For example, some MWDs manufacturers suggest the replacement of the filter once a year; others suggest a replacement every six months with a general prediction that the lifetime of the filter is around 2700 L or one year. Based on these indications, filter replacement in the MWDs with low water consumption can sometimes be delayed resulting in a loss in the efficiency of dispensers, which can create an ideal habitat for bacterial growth and proliferation. The manuals did not report specific instructions about the need for microbiological or chemical control, i.e., a method to check the water quality. After completing the microbiological analysis and identifying the non-conformities, it is essential to adopt maintenance and sanitization procedures using appropriate disinfectants, considering the concentrations and contact times required. These procedures must be performed by trained personnel, as established by the Ministerial Decree n. 25 (7 February 2012) [5] and the effectiveness of the actions undertaken must be verified with analytical checks within a short time of the execution of the intervention itself.

## 5. Conclusions

The need for good quality water, both organoleptically and microbiologically, has led to the development of devices with new technologies to satisfy consumer demand. Our findings provide insight into the need for MWDs to be sold with correct and clear manuals explaining the cleaning procedures with specific instructions about flushing the input and output of the device, cleaning of nozzles, and the need for long-term procedures such as changing the filters and microbial analyses periodically performed in line with regulations on microbiological control. These considerations could help manufacturers improve the devices, the consumers to adopt good practices during the use of MWDs, and the public health authorities to demand compliance with water quality regulation.

## Figures and Tables

**Table 1:** Contamination of total of samples of MWDs (microfiltered water dispensers, *n* = 46) and contamination at the sampling point (input and output): HPCs (heterotrophic plate counts) at 36 °C, HPCs at 22 °C, Pseudomonas aeruginosa and other pathogenic microorganisms (Enterococci, *Escherichia coli*, and *Staphylococcus aureus*).

Sampling Point	Type of Analysis	Positive Samples	Positive Samples (%)	Log cfu/mL ± SD	Log cfu/100 mL ± SD	Reference Value *
Positive Samples *n* = 93	HPCs at 36 °C	73/93	78.49	2.62 ± 0.64		1.3 Log cfu/mL
	HPCs at 22 °C	34/93	36.56	3.00 ± 0.50		2 Log cfu/mL
	*Pseudomonas aeruginosa*	20/93	21.51		1.48 ± 0.80	0 Log cfu/100 mL
	Other pathogenic microorganisms (Enterococci, *Escherichia coli, and Staphylococcus aureus*)	3/93	3.23		0.49 ± 0.20	0 Log cfu/100 mL
Input Water *n* = 93	HPCs at 36 °C	41/93	44.09	2.13 ± 0.50		1.3 Log cfu/mL
	HPCs at 22 °C	6/93	6.45	2.66 ± 0.56		2 Log cfu/mL
	*Pseudomonas aeruginosa*	0/93	ND		ND	0 Log cfu/100 mL
	Others pathogenic microorganisms (Enterococci, *Escherichia coli, and Staphylococcus aureus*)	0/93	ND		ND	0 Log cfu/100 mL
Output Water *n* = 93	HPCs at 36 °C	56/93	60.22	2.90 ± 0.65		1.3 Log cfu/mL
	HPCs at 22 °C	33/93	35.48	3.06 ± 0.60		2 Log cfu/mL
	*Pseudomonas aeruginosa*	20/93	21.51		1.48 ± 0.80	0 Log cfu/100 mL
	Other pathogenic microorganisms (Enterococci, *Escherichia coli, and Staphylococcus aureus*)	3/93	3.23		0.49 ± 0.20	0 Log cfu/100 mL

* Italian regulation for drinking water: Legislative Decree n. 31 (02.02.2001) [19]; ND: Non-Detected

**Table 2:** Total contamination of total samples of MWDs of Type A and B with respect to the sampling point (input and output): HPCs at 36 °C, HPCs at 22 °C, *Pseudomonas aeruginosa*, and other pathogenic microorganisms (Enterococci, *Escherichia coli*, and *Staphylococcus aureus*).

Type of MWDs	Type of Analysis	Contamination of Total of Samples				Contamination of Input Water				Contamination of Output Water				Reference Value *
		Positive Samples	Positive Samples (%)	Log cfu/mL ± SD	Log cfu/100 mL	Positive Samples	Positive Samples (%)	Log cfu/mL ± SD	Log cfu/ 100 mL	Positive Samples	Positive Samples (%)	Log cfu/mL ± SD	Log cfu/100 mL	
Type A (*n* = 11)	HPCs at 36 °C	20/39	51.28	2.33 ± 0.56		17/39	43.59	2.16 ± 0.47		10/39	25.64	2.67 ± 0.38		1.3 Log cfu/mL
	HPCs at 22 °C	6/39	15.38	2.51 ± 0.44		2/39	5.13	2.86 ± 0.28		5/39	12.82	2.58 ± 0.62		2 Log cfu/mL
	*Pseudomonas aeruginosa*	1/39	2.56		0.90	0/39	ND		ND	1/39	2.56		0.90	0 Log cfu/100 mL
	Others pathogenic microorganisms (Enterococci, *Escherichia coli, Staphylococcus aureus*)	0/39	ND			0/39	ND		ND	0/39	ND		ND	0 Log cfu/100 mL
Type B (*n* = 35)	HPCs at 36 °C	53/54	98.15	2.72 ± 0.68		24/54	44.44	2.11 ± 0.53		46/54	85.19	2.97 ± 0.67		1.3 Log cfu/mL
	HPCs at 22 °C	28/54	51.85	3.10 ± 0.49		4/54	7.41	2.56 ± 0.68		28/54	51.85	3.14 ± 0.56		2 Log cfu/mL
	*Pseudomonas aeruginosa*	19/54	35.19		1.51 ± 0.81	0/54	ND		ND	19/54	35.19		1.51 ± 0.81	0 Log cfu/100 mL
	Others pathogenic microorganisms (Enterococci, *Escherichia coli, Staphylococcus aureus*)	3/54	5.56		0.49 ± 0.20	0/54	ND		ND	3/54	5.56		0.49 ± 0.20	0 Log cfu/100 mL

* Italian regulation for drinking water: Legislative Decree n. 31 (02.02.2001) [19]. ND: Non-Detected.

**Table 3:** Regular and implemented procedures in Type A and Type B MWDs.

	Regular Procedures	Implemented Procedures during the Study
**Type A**	Changing the filter and lamp once a year Complete cleaning every six months using hydrogen peroxide solution (3% *v/v* for 10 min)	External daily cleaning of MWDs Daily cleaning of supply point with descaling solution Flushing input and output points (with 0.5 L water once a week), especially after the weekend or the holidays Cleaning the glass UV lamp once every six months Inner UV lamp substitution every 9000 h or once a year UV lamp, at supply point, substitution every 6000 h or every six months Complete cleaning every six months using hydrogen peroxide solution (3% *v/v* for 30 min).
**Type B**	Changing the filter and lamp, once a year Complete cleaning every six months using hydrogen peroxide solution (3% *v/v* for 10 min)	Flushing the input and output (with 1 L water once a week), after the weekend or the holidays Daily cleaning of supply point Complete cleaning every six months using hydrogen peroxide solution (3% *v/v* for 30 min) UV lamp, at supply point, substitution every 6000 h or every six months.

## References

[1] Liguori G., Cavallotti I., Arnese A., Amiranda C., Anastasi D., Angelillo I.F. (2010). Microbiological quality of drinking water from dispenser in Italy. BMC Microbiol..

[2] Marzano M.A., Balzaretti C.M. (2001). Preliminary investigation on the bacteriological quality of microfiltered drinking water dispensers in catering establishments. Ital. J. Food Saf..

[3] Siddiqi K.S., Husen A., Rao R.A.K. (2018). A review on biosynthesis of silver nanoparticles and their biocidal properties. J. Nanobiotechnol..

[4] Das R., Gang S., Nath S.S. (2011). Preparation and antibacterial activity of silver nanoparticles. J. Biomater. Nanobiotechnol..

[5] Ministerial Decree 07.02.2012, n. 25. Technical Provisions Concerning Equipment for the Treatment of Water for Human Consumption. OJ General Series n. 69 of 22.03.2012. (Decreto Ministeriale 07.02.2012, n. 25. Disposizioni Tecniche Concernenti Apparecchiature Finalizzate al Trattamento Dell’acqua Destinata al Consumo Umano. G.U. Serie Generale n. 69 del 22.03.2012). https://www.physico.eu/pdf/d-m-7-febbraio-2012-n-25.pdf.

[6] Garvey M., Rabbitt D., Rowan A.N. (2015). Pulsed ultraviolet light inactivation of *Pseudomonas aeruginosa* and *Staphylococcus aureus* biofilms. Water Environ. J..

[7] Okpara C.G., Oparaku N.F., Ibeto C.N. (2001). An overview of water disinfection in developing countries and potentials of renewable energy. J. Environ. Sci. Technol..

[8] Tingpej P., Tiengtip R., Kondo S. (2015). Decontamination efficacy of ultraviolet radiation against biofilms of common nosocomial bacteria. J. Med. Assoc..

[9] Rutala W.A., Weber D.J. (2011). Are room decontamination units needed to prevent transmission of environmental pathogens. Infect. Control Hosp. Epidemiol..

[10] Walker J.T., Marsh P.D. (2007). Microbial biofilm formation in DUWS and their control using disinfectants. J. Dent..

[11] Douterelo I., Jackson M., Solomon C., Boxall J. (2016). Microbial analysis of in situ biofilm formation in drinking water distribution systems: Implications for monitoring and control of drinking water quality. Appl. Microbiol. Biotechnol..

[12] Momba M.N.B., Kfir R., Venter S.N., Cloete T.E. (2000). An overview of biofilm formation in distribution systems and its impact on the deterioration of water quality. Water SA.

[13] Nelson K.Y., McMartin D.W., Yost C.K., Runtz K.J., Ono T. (2013). Point of use water disinfection using UV light-emitting diodes to reduce bacterial contamination. Environ. Sci. Pollut. Res. Int..

[14] Szymańska J. (2006). Bacterial decontamination of DUWL biofilm using Oxygenal 6. Ann. Agric. Environ. Med..

[15] Costerton J.W., Stewart P.S., Greenberg E.P. (1999). Bacterial biofilms: A common cause of persistent infections. Science.

[16] Oliveira N.M., Martinez-Garcia E., Xavier J., Durham W.M., Kolter R., Wook K., Foster K.R. (2015). Biofilm Formation as a Response to Ecological Competition. PLoS Biol..

[17] Stewart P.S., Costerton J.W. (2001). Antibiotic resistance of bacteria in biofilms. Lancet.

[18] Roya R., Tiwaria M., Donelli G., Tiwaria V. (2018). Strategies for combating bacterial biofilms: A focus on anti-biofilm agents and their mechanisms of action. Virulence.

[19] Legislative Decree 02.02.2001, n.31. Implementation of the Water Quality Directive 98/83/EC Relative to Water Quality Intended for Human Consumption. OJ. of the Italian Republic n. 52, 3.03.2001. (Decreto Legislativo 02.02.2001, n. 31. Attuazione Della Direttiva 98/83/CE Relativa alla Qualità delle Acque Destinate al Consumo Umano. G.U. della Repubblica Italiana n. 52, 3.03.2001). http://www.camera.it/parlam/leggi/deleghe/01031dl.htm.

[20] European Union (1998). Council Directive 98/83/EC of 3 November 1998 on the quality of water intended for human consumption. Off. J. Eur. Communities.

[21] Baumgartner A., Grand M. (2006). Bacteriological quality of drinking water from dispensers (Coolers) and possible control measure. J. Food Prot..

[22] Guidelines on Water Treatment Devices Spent on Human Consumption under the D.M. 7 February 2012, n. 25. http://www.salute.gov.it/imgs/C_17_pubblicazioni_1946_allegato.pdf.

[23] Chaberny I.F., Kaiser P., Sonntag H.G. (2006). Can soda fountains be recommended in hospitals?. Int. J. Hyg. Environ. Health.

[24] Lévesque B., Simard P., Gauvin D., Gingras S., Dewailly E., Letarte R. (1994). Comparison of the microbiological quality of water coolers and that of municipal water systems. Appl. Environ. Microbiol..

[25] Zanetti F., De Luca G., Sacchetti R. (2009). Control of bacterial contamination in microfiltered water dispensers (MWDs) by disinfection. Int. J. Food Microbiol..

[26] Regulation (EC) n. 852/2004 of the European Parliament and of the Council 29.04.2004, article 6, on the Hygiene of Foodstuffs (GU l 139 of 30.4.2004, pag 1) (Regolamento (CE) n. 852/2004 del Parlamento Europeo e del Consiglio 29.04.2004, articolo 6, Sull’igiene dei Prodotti Alimentari (GU l 139 del 30.4.2004, pag. 1)). https://www.certifico.com/chemicals/legislazione-chemicals/264-legislazione-chemicals-food/4288-regolamento-ce-n-852-2004.

[27] World Health Organization Guidelines for Drinking-Water Quality. http://apps.who.int/iris/bitstream/10665/44584/1/9789241548151_eng.pdf.

[28] EN ISO 19458:2006—Water Quality—Sampling for Microbiological Analysis. https://www.iso.org/standard/33845.html.

[29] UNI EN ISO 6222:2001—Water Quality—Enumeration of Culturable Micro-Organisms—Colony Count by Inoculation in a Nutrient Agar Culture Medium, Geneva: International Organization for Standerdization. http://store.uni.com/catalogo/index.php/uni-en-iso-6222-2001.html.

[30] ISO 7899-2:2000 Water Quality—Detection and Enumeration of Intestinal Enterococci—Part 2: Membrane Filtration Method. https://onlinelibrary.wiley.com/doi/full/10.1111/wej.12088.

[31] UNI EN ISO 16266:2008—Water Quality—Detection and Enumeration of Pseudomonas aeruginosa—Method by Membrane Filtration. http://store.uni.com/catalogo/index.php/uni-en-iso-16266-2008.html?josso_back_to=http://store.uni.com/josso-security-check.php&josso_cmd=login_optional&josso_partnerapp_host=store.uni.com.

[32] UNI EN ISO 9308-1:2017 Water Quality—Enumeration of Escherichia coli and Coliform Bacteria—Part 1: Membrane Filtration Method for Waters with Low Bacterial Background Flora. http://store.uni.com/catalogo/index.php/uni-en-iso-9308-1-2017.html.

[33] ISO 16140-2:2016 Microbiology of the Food Chain—Method validation—Part 2: Protocol for the Validation of Alternative (Proprietary) Methods against a Reference Method. https://www.iso.org/standard/54870.html.

[34] Wauters G., Boel A., Voorn G.P., Verhaegen J., Meunier F., Janssens M., Verbist J.L. (1995). Evaluation of a new identification system, Crystal Enteric/Non-Fermenter, for gram-negative bacilli. J. Clin. Microbiol..

[35] Ministerial Decree 14.06.2017. Implementation of Directive (EU) 2015/1787 Amending Annexes II and III of Directive 98/83/EC on the Quality of Water Intended for Human Consumption. Amendments to Annexes II and III of Legislative Decree 2 February 2001, n. 31. (17A05618) (GU General Series n.192 of 18-08-2017) (Decreto Ministeriale 14.06.2017 Recepimento Della Direttiva (UE) 2015/1787 che Modifica gli Allegati II e III Della Direttiva 98/83/CE Sulla Qualità Delle Acque Destinate al Consumo Umano. Modifica Degli Allegati II e III del decreto legislativo 2 febbraio 2001, n. 31. (17A05618) (GU Serie Generale n.192 del 18-08-2017). https://www.google.com/url?sa=t&rct=j&q=&esrc=s&source=web&cd=3&ved=2ahUKEwis_Nm09ZTfAhUKw4sKHRPbAFAQFjACegQIAhAC&url=http%3A%2F%2Fwww.ceirsa.org%2Ffd.php%3Fpath%3D201710%2FDM_14_06_2017Acque_destinate_al_consumo_umano.pdf&usg=AOvVaw3eguX4JKnVJArmnYdE1oVD.

[36] Commission Directive (EU) 2015/1787 of 06.10.2015 Amending Annexes II and III to Council Directive 98/83/EC on the Quality of Water Intended for Human Consumption. https://eur-lex.europa.eu/legal-content/EN/TXT/?uri=uriserv%3AOJ.L_.2015.260.01.0006.01.ENG.

[37] Legislative Decree 09.04.2008, n.81. Implementation of Article 1 of the Law of 3 August 2007, n. 123, Concerning the Protection of Health and Safety in the Workplace. OJ. General Series n.101 of 30-04-2008—Suppl. Ordinary n. 108 (Decreto Legislativo 09.04.2008, n. 81. Attuazione dell’articolo 1 della legge 3 agosto 2007, n. 123, in Materia di Tutela Della Salute e Della Sicurezza nei Luoghi di Lavoro. GU Serie Generale n.101 del 30-04-2008—Suppl. Ordinario n. 108). https://community.intelex.com/library/peer-resources/italy-legislative-decree-9-april-2008-n-81-1.

[38] Zannetti F., de Luca G., Leoni E., Sacchetti R. (2014). Occurrence of non-fermenting gram negative bacteria in drinking water dispensed from point-of-use microfiltration devices. Ann. Agric. Environ. Med..

[39] Lubart R., Lipovski A., Nitzan Y., Friedmann H. (2011). A possible mechanism for the bactericidal effect or visible light. Laser Ther..

[40] Kuo J., Chen C., Nellor M. (2003). Standardized Collimated Beam Testing Protocol for Water/Wastewater Ultraviolet Disinfection. J. Environ. Eng..

[41] Zlatanovic L., van der Hoek J.P., Vreeburg J.H.G. (2017). An experimental study on the influence of water stagnation and temperature change on water quality in a full-scale domestic drinking water system. Water Res..

[42] Amanidaz N., Zafarzadeh A., Mahvi A.H. (2015). The Interaction between Heterotrophic Bacteria and Coliform, Fecal Coliform, Fecal Streptococci Bacteria in the Water Supply Networks. Iran. J. Public Health.

[43] Lin S., Svoboda K., Giletto A., Seibert J., Puttaiah R. (2011). Effects of hydrogen peroxide on dental unit biofilms and treatment water contamination. Eur. J. Dent..

[44] West A., Tesla P., Lineback C., Oliver H. (2018). Strain, disinfectant, concentration and contact time quantitatively impact disinfectant efficacy. Antimicrob. Resist. Infect. Control.

